# Postgraduate medical education in Japan: Missed opportunity for learning clinical reasoning

**DOI:** 10.1002/jgf2.202

**Published:** 2018-09-02

**Authors:** Yasuharu Tokuda, Mano Soshi, Tomoya Okubo, Yuji Nishizaki

**Affiliations:** ^1^ Muribushi Okinawa for Teaching Hospitals Okinawa Japan; ^2^ Minami Seikyo Hospital Aichi Japan; ^3^ Research Division The National Center for University Entrance Examinations Tokyo Japan; ^4^ Medical Technology Innovation Center Juntendo University Tokyo Japan; ^5^ Clinical Research and Trial Center Juntendo University Hospital Tokyo Japan

Most Japanese physicians who graduated before 2004 received training in only single specialty during their residency and thus lacked attitudes, skills, and knowledge for basic primary and emergency care.^1^ Some of them revealed inadequate quality of primary and emergency care and public pressure throughout Japan had promoted the introduction of mandatory medical education for all newly graduating physicians.^2^

Consequently, the current postgraduate medical education (PGME) was implemented in 2004 by the Japanese government as new 2‐year super‐rotation program, including internal medicine, emergency medicine, and community medicine as mandatory rotations and other specialties as selective rotations.^3^ Objectives of this PGME are to educate residents to acquire attitudes, skills, and knowledge for providing basic clinical care. A study showed that physicians who received the current PGME program were more likely to adhere to an appropriate standard of care in emergency medicine and were more confident in caring for patients with acute illnesses, compared to those who graduated before 2004 and did not receive the current PGME.^4^

Although the current PGME is better than the no‐rotation training prior to 2004, there may be still some weaknesses of the current PGME. First, overtesting in diagnostic process is highly prevalent in Japanese hospitals,^5^ and thus, some residents, if not all, may tend to order a long list of diagnostic tests, which may mask lack of their diagnostic reasoning skills. Because overtesting can cause harm to patients through downstream tests including invasive tests, the overtesting practice might potentially lead to greater harmful events in the future of Japan. Second, some residents in Japan have interests in acquiring procedural skills, which can obviously show their competency to other members of their care team, including teaching faculty. However, competent residents need not only procedural skills, but also basic skills of clinical reasoning based on good history taking and physical examination.

Therefore, we conducted a nationwide study comparing postgraduate year (PGY)‐1 and PGY‐2 residents for knowledge of basic clinical care, including clinical reasoning, based on results of general medicine in‐training examination (GM‐ITE). The previous results of this examination have been published elsewhere and elucidated important educational factors related to the GM‐ITE scores.^6^, ^7^, ^8^, ^9^ We analyzed the most recent (February 2017) GM‐ITE scores of resident physicians between postgraduate year (PGY)‐1 and PGY‐2 resident physicians in 459 Japanese teaching hospitals which participated in the GM‐ITE.

A total of 5593 resident physicians (2678 PGY‐1 and 2915 PGY‐2) participated in the examination. A total of 491 residents were excluded from analyses, because we could not obtain their consent to the data use. Overall mean score among PGY‐1 and PGY‐2 were 32.3 ± 5.1 and 32.5 ± 5.5, respectively, and these were not significantly different. However, additional analysis was conducted using scores of residents only in top 200 hospitals in terms of the higher mean scores among participating residents. This subgroup analysis showed mean score 34.9 ± 5.0 of PGY‐2 was significantly higher than that 34.4 ± 4.7 of PGY‐1 (Table 1).

**Table 1:** Comparison of GM‐ITE scores between PGY‐1 and PGY‐2

PGY	GM‐ITE score	*P*‐value
Residents in top 200 hospitals (n = 2413)		
PGY‐1	34.4 ± 4.7	0.03^a^
PGY‐2	34.9 ± 5.0	
All residents (n = 5102)		
PGY‐1	32.3 ± 5.1	>0.05
PGY‐2	32.5 ± 5.5	

GM‐ITE, general medicine in‐training examination; PGY, postgraduate year.

a Statistically significant.

Based on our results, there are few improvements of the basic clinical knowledge over the PGY‐1 to PGY‐2 years in low‐score hospitals, but residents in high‐score hospitals have the significant improvement. In other words, good teaching hospitals may effectively teach knowledge for clinical reasoning to residents, but other hospitals may not. Our result should be an alarm for teachers and residents in Japan, and further studies for assessing clinical reasoning skills in each PGY year of Japanese residents should be considered. Meanwhile, clinician educators in teaching hospitals should focus more on teaching clinical reasoning.

## CONFLICT OF INTEREST

The authors have stated explicitly that there are no conflicts of interests in connection with this article.
